# Stem cell therapy for the treatment of early stage avascular necrosis of the femoral head: a systematic review

**DOI:** 10.1186/1471-2474-15-156

**Published:** 2014-05-16

**Authors:** Rick L Lau, Anthony V Perruccio, Heather MK Evans, Safiyyah R Mahomed, Nizar N Mahomed, Rajiv Gandhi

**Affiliations:** 1 Nickle 3, Kingston General Hospital, 76 Stuart Street, Kingston, ON K7L 2 V7, Canada; 2 Division of Orthopaedic Surgery, Toronto Western Hospital, 399 Bathurst Street EW 1-427, Toronto, Ontario M5T 2S8, Canada

**Keywords:** Avascular necrosis, Femoral head, Stem cells, Treatment, Precollapse

## Abstract

**Background:**

Avascular necrosis (AVN) of the femoral head (FH) is believed to be caused by a multitude of etiologic factors and is associated with significant morbidity in younger populations. Eventually, the disease progresses and results in FH collapse. Thus, a focus on early disease management aimed at joint preservation by preventing or delaying progression is key. The use of stem cells (SC) for the treatment of AVN of the FH has been proposed. We undertook a systematic review of the medical literature examining the use of SC for the treatment of early stage (precollapse) AVN of the FH, in both pre-clinical and clinical studies.

**Methods:**

Data collected included: Pre-clinical studies – model of AVN, variety and dosage of SC, histologic and imaging analyses. Clinical studies – study design, classification and etiology of AVN, SC dosage and treatment protocol, incidence of disease progression, patient reported outcomes, volume of necrotic lesion and hip survivorship.

**Results:**

In pre-clinical studies, the use of SC uniformly demonstrated improvements in osteogenesis and angiogenesis, yet source of implanted SC was variable. In clinical studies, groups treated with SC showed significant improvements in patient reported outcomes; however hip survivorship was not affected. Discrepancies regarding dose of SC, AVN etiology and disease severity were present.

**Conclusions:**

Routine use of this treatment method will first require further research into dose and quality optimization as well as confirmed improvements in hip survivorship.

## Background

Avascular necrosis (AVN) of the femoral head (FH) is a debilitating and painful disease with multiple etiologic risk factors [1-4]. These include, but are not limited to, corticosteroid use [1,3-6], alcohol abuse [1-4], previous trauma [1,3], hemoglobinopathy [7], Gaucher’s disease and coagulopathies [8]. The onset of AVN may also be idiopathic [7]. AVN of the FH most commonly affects younger or middle aged adults [2,9,10]. Disease progression commonly leads to collapse of the affected FH and ultimately, development of osteoarthritis [1,3,4,7,11,12]. Outcomes of THA for these younger and more active patients have been poor, primarily due to the limited lifetime and durability of total hip arthroplasty (THA) [6,10,11]. As a result, there has been an increased focus on early-interventions for AVN, aimed at preservation of the native articulation [6,12]. Core decompression (CD) is currently the most widely accepted treatment for early-stage AVN of the FH; however, due to limited efficacy, its use has been debated [12]. The development of safe, cost-effective, and potentially minimally invasive joint preserving treatments for early stage (precollapse) AVN merits further investigation.

Several studies, both clinical and pre-clinical, have demonstrated the efficacy of stem cells (SC) for the treatment of AVN of the FH [1,3,11,13-15]. SC can be obtained from a variety of sources, including autologous bone marrow [2,4,10,11,16,17], adipose tissue [10] and dental-pulp [4]. SC have been shown to promote bone formation [4,6,11,12] and neovascularization [6,11] in vitro. Additionally, patients treated with SC in conjunction with CD demonstrated significant improvements in Harris Hip Scores (HHS) [15] as well as decreased hip pain and symptoms compared to those treated with CD alone [2]. Yan et al. documented that stem cells implanted into the necrotic FH not only survive, but thrive and proliferate [15]. Although the pathogenesis of AVN is unclear [17] many hypothesize that SC work to improve early stage AVN potentially as a function of their critical role in the regulation and improvement of osteogenesis and angiogenesis [11,12]. Furthermore, it is thought that mesenchymal SC implanted into the necrotic FH may differentiate into osteoblasts or vascular endothelial cells, thereby promoting bone repair and regeneration [12]. Despite encouraging results in preclinical (basic science) and clinical studies, improvements in hip survivorship or time to THA has not been uniformly reported and remains controversial [2,8,14].

The purpose of our study was to perform a systematic review of the current medical literature on the treatment of early stage AVN of the FH using SC implanted via CD. We examined both preclinical studies and clinical studies. We reported bone healing outcomes (histologic and imaging outcomes) from preclinical papers and all examined outcomes from available clinical papers.

## Methods

### Eligibility criteria

Manuscripts were deemed eligible for our review if they evaluated treatment of early stage AVN of the FH with SC implanted via CD. We defined early stage AVN as precollapse of the FH. Both clinical and preclinical manuscripts were selected. For clinical trials, we included studies on patients age > 18. All types of clinical studies were eligible for inclusion to this review. Studies of all languages were eligible for inclusion to this review. For studies reporting on the same group of patients at multiple follow up periods, the most recent publication was used in this review. For preclinical studies, manuscripts were eligible if they examined bone healing either histologically, or by imaging techniques. If studies examined other treatments such as vascularized fibular grafting or bone morphogenic proteins, they were excluded unless the data on SC and CD were presented separately from the other treatments, to allow us to examine the effect of SC specifically.

### Study identification

A systematic, computerized search for potential manuscripts was performed by three independent reviewers (HE, SM, RL). Pubmed (−July 2012), Ovid Medline (−July 2012) and EMBASE (−July 2012) databases were used to identify studies. Key words used for the search were: AVN or avascular necrosis or osteonecrosis AND stem cells; AVN or avascular necrosis or osteonecrosis AND autologous bone marrow. Abstracts were retrieved for all manuscripts considered relevant by title. Abstracts were independently reviewed and any disagreements were resolved by discussion. Full length articles of relevant abstracts were reviewed for inclusion. Bibliographies of the full length articles were also searched for other potential studies and full length articles were retrieved.

Outcomes data were extracted by two reviewers (HE, RL) using prearranged summary tables. Data extraction for preclinical studies included study design, animal model, type of SC used, sample size, and outcomes measured. For the clinical studies, study type, sample size, potential biases, AVN classification, AVN etiology, SC dose and cell type, and outcomes measured were recorded.

## Results

### Study selection

**Figure 1:**
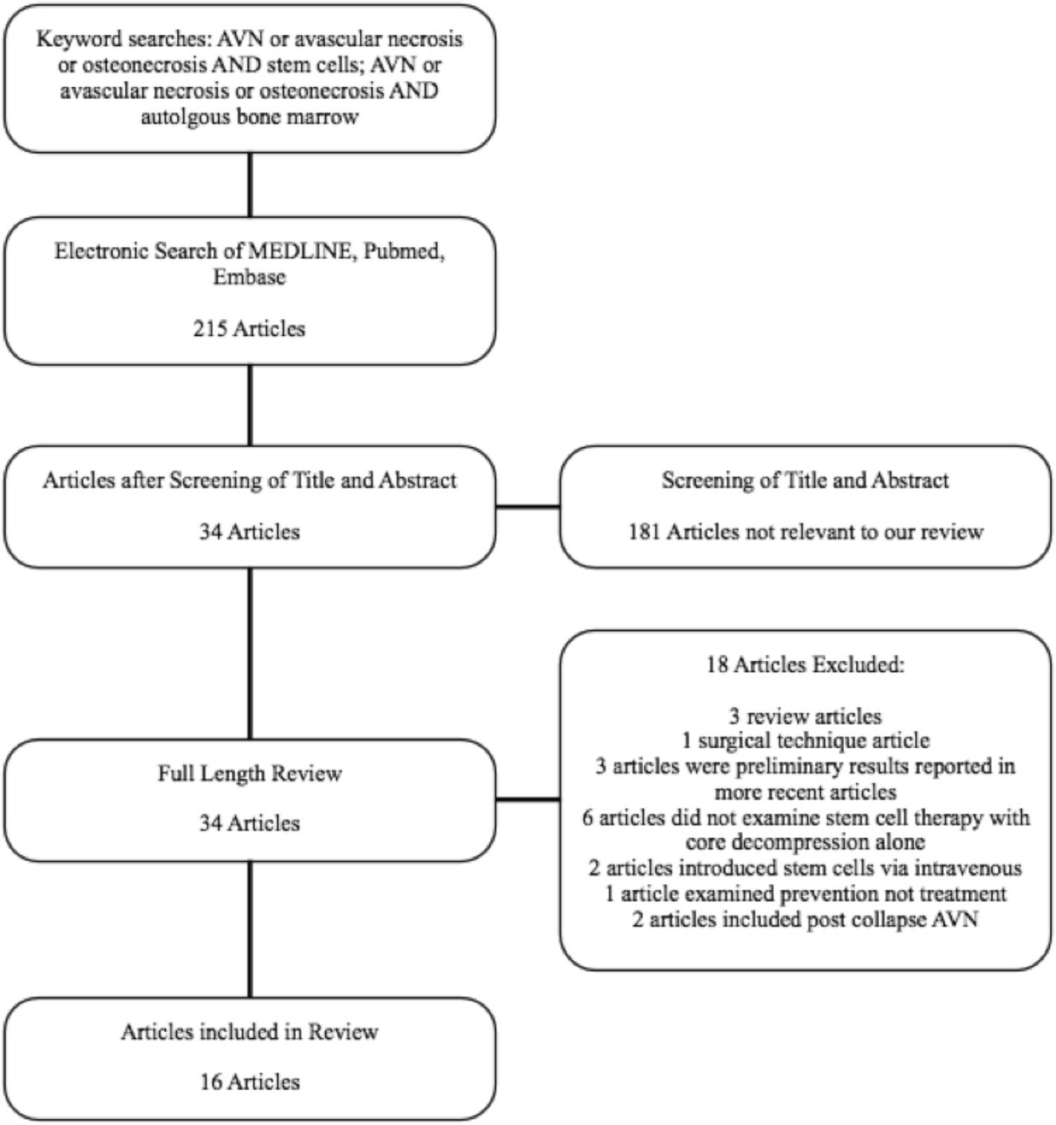
Search, screening and selection of articles for review.

We identified 215 abstracts using our electronic search. Thirty-four met the initial screening inclusion criteria and the full-length articles were retrieved and reviewed. Following the full-length reviews, 16 studies (11 preclinical, 5 clinical) met our inclusion criteria and were retained for this review. Eighteen studies were excluded: 3 were review articles [18-20], one was a surgical technique article [21], 3 articles were early/pilot results which were subsequently included in future articles [7,16,22], 6 articles examined SC treatment in conjunction with therapies other than CD (i.e. vascularized fibula) [23-28], two articles used SC transplanted intravenously instead of by CD [13,29], 1 article examined prevention of AVN not treatment [5], and two articles included patients with post collapse AVN of the FH [1,15] (Figure 1).

### Preclinical study characteristics

#### Study design

All preclinical studies utilized an animal model (8 rabbit [6,10,17,30-34], 2 dog [11,12] and 1 sheep model [4]). AVN was induced using methylprednisolone injection in 6 of the 11 studies [6,10,30-32,34]. One model utilized liquid nitrogen to create AVN of the FH [17]. Three studies utilized a femoral neck osteotomy to induce AVN [11,12,33]. One study induced AVN by instillation of ethanol to the FH via CD [4].

#### Stem cell treatment

The type of SC implanted were variable yet may be classified into three groups: 1) bone marrow derived stem cells – concentrated, un-cultured bone marrow mononuclear cells (BMMNC), fresh (1 study) [6] or cryopreserved (1 study) [32], and mesenchymal stem cells obtained post BMMNC culture (4 studies) [11,12,30,33]; 2) adipose derived stem cells (2 studies) [10,34]; 3) other – peripheral blood hematopoietic SC (1 study) [17], cultured endothelial progenitor cells (EPC) (1 study) [31], and human immature dental pulp SC (1 study) [4] (Table 1).

**Table 1:** Preclinical study characteristics and results

**Study**	**Animal model**	**Stem cell therapy**	**Results**					
			**Histology**		**Imaging**			
			**Bone formation**	**Angiogenesis**	**X-ray**	**SPECT**	**CT**	**MR**
Wen et al. [30]	Rabbit	BMMNC-MSC	+ve	+ve*	--	--	+ve	+ve
Sun et al. [6]	Rabbit	BMMNC	+ve*	+ve*	--	--	+ve*	--
Yan et al. [12]	Dog	BMMNC-MSC	+ve*	--	--	--	--	--
Feitosa et al. [4]	Sheep	hIDPSC, BMMNC-MSC	+ve	--	--	--	--	--
Abudusaimi et al. [10]	Rabbit	ADSC	+ve	--	--	--	+ve*	--
Aimaiti et al. [34]	Rabbit	ADSC	+ve	+ve	--	--	+ve*	--
Song et al. [17]	Rabbit	PBSC	+ve	+ve*	--	--	--	--
Sun et al. [31]	Rabbit	EPC	+ve*	+ve*	--	--	--	--
Hang et al. [11]	Dog	BMMNC-MSC	+ve*	+ve*	+ve	+ve	--	--
Wen et al. [33]	Rabbit	BMMNC-MSC	+ve	--	--	--	--	--
Xie et al. [32]	Rabbit	cBMMNC	+ve*	+ve*	--	--	+ve*	--

BMMNC-MSC = mesenchymal stem cells obtained post bone marrow mononuclear cell culture, hIDPSC = human immature dental pulp stem cells, ADSC = adipose derived stem cells, PBSC = peripheral blood stem cells, EPC = endothelial progenitor cells, cBMMNC = cryoperserved bone marrow mononuclear cells, +ve = positive effect compared to controls, −ve = negative effect compared to controls, -- = not measured, * = statistically significant (p < 0.05).

#### Histologic analysis

All preclinical studies performed a histologic evaluation of the FH’s after SC treatment, examining for osteogenesis. In addition, 5 of the studies performed a quantitative histomorphometric analysis for osteogenesis [6,11,12,31,32]. Seven studies examined the FH’s for neovascularization [6,11,17,30-32,34].

#### Imaging analysis

Six of the 11 studies used a variety of imaging techniques to evaluate bone formation [6,10,11,30,32,34]. Wen et al. used computed tomography (CT), magnetic resonance imaging (MRI), and CT perfusion imaging to evaluate the effectiveness of autologous cultured bone marrow SC therapy [30]. Abudusaimi et al., Xie et al. and Aimaiti et al. used micro CT scan to evaluate bone volume, bone mineral density and trabecular volume [10,32,34]. Sun et al. used micro CT angiography to evaluate angiogenesis [6]. Hang et al. used single photon emission computed tomography (SPECT) and radiographs to evaluate bone density and FH shape [11].

### Preclinical study findings

#### Histology (11 studies)

Nine studies reported an increase in bone formation in the SC treatment group compared to CD alone [4,6,10-12,31-34] and two studies reported an increase in bone formation compared to no treatment [17,30]. In the 5 studies that performed a quantitative histomorphometric analysis, all 5 studies reported significantly increased bone formation (p < 0.05) following SC therapy versus CD alone [6,11,12,31,32].

Five studies performing a histologic assessment of neovascularization following SC treatment found improvement (p < 0.05) in the SC treatment groups compared to CD alone [6,11,30-32]. One study found higher levels of neovascularization compared to no treatment [17]. One study did not perform statistical analysis but reported higher levels of neovascularization [34] (Table 1).

#### Imaging (6 studies)

On CT imaging, Wen et al. found that the SC group had imaging that closely resembled the normal hip in comparison to CD alone [30]. On MRI imaging they found decreased FH necrosis volume compared to no treatment, and on CT perfusion imaging they found blood volume to be similar to normal hips (no statistics reported) [30]. Abudusaimi et al., Xie et al. and Aimaiti et al. found significantly higher bone volume, bone mineral density and trabecular volume in the SC group compared to CD alone (p < 0.05) on micro CT [10,32,34].

Sun et al. performed micro CT angiography and found improved angiogenesis (p < 0.05) [6]. Hang et al. found that the CD only group had decreased uptake of radioactivity on SPECT compared to SC treatment group, indicative of worse perfusion [11]. Radiographs also revealed that the CD only group had worse results, with irregular articular surfaces and heterogenous density of the FH in comparison to the SC group (no statistics reported) [11] (Table 1).

### Clinical study characteristics

#### Study design

The clinical studies included two randomized control trials (RCT) [8,14], one non randomized comparison study (nRCT) [2], and two case series [9,35]. The length of follow up for the RCTs was 24 months [14], and 60 months [8]. The nRCT had a follow up of 60 months [2]. The case series’ examined outcomes at an average follow up of 27.6 months (12–40 months) [9], and 13 years (8–18 years) [35].

#### Sample size

Sample size (n) was recorded as number of hips. Combined, the 5 studies comprised 763 hips. Across studies, n varied from a minimum of 24 [2] to a maximum of 534 [35] (Table 2). None of the studies reported a power calculation.

**Table 2:** Clinical Study Characteristics

**Study**	**Type**	**n**	**Initial AVN class**	**AVN etiology (# of hips)**	**Treatment protocol**
Hernigou et al. [35]	Case series	534	Ficat 1-2	steroid (101), ethanol (150), sickle cell (166), not specified (117)	Concentrated BMMNC
Wang et al. [9]	Case series	50	ARCO 1-2	steroid (25), ethanol (19), idiopathic (6)	Concentrated BMMNC
Gangji et al. [2]	nRCT	24	ARCO 1-2	steroid (20), ethanol (2), idiopathic (2)	Concentrated BMMNC
Sen et al. [14]	RCT	51	ARCO 1-2	trauma (17), steroid (20), ethanol 8), idiopathic (2), pregnancy (2), Cushings (2)	Concentrated BMMNC
Zhao et al. [8]	RCT	97	ARCO 1C-2C	trauma (20), steroid (24), ethanol (19), Caisson (11), idiopathic (30) *(some patients had more than one etiology)*	Cultured BMMNC

BMMNC = bone marrow mononuclear cells, n = number of hips, ARCO = Association Research Circulation Osseous classification, nRCT = non randomized control trial, RCT = randomized control trial.

#### Avascular necrosis classification and etiology

Combined, the studies included patients with the following etiologies for AVN: trauma [8,14], steroid use [2,8,9,14,35], ethanol use [2,8,9,14,35], pregnancy [14], Cushing’s disease [14], Caisson disease [8], sickle cell disease [34], and idiopathic [2,8,9,14]. Hernigou et al. did not specify the cause of AVN for 117 of 534 hips in their case series [35] (Table 2). The severity of AVN was classified using two grading systems across the 5 studies. Four of the studies utilized the Association Research Circulation Osseous (ARCO) classification [2,8,9,14] and included patients with an ARCO classification of 1–2 (precollapse). One study used the Ficat classification, and included patients in Ficat stage 1 or 2 [35] (Table 2).

#### Stem cell treatment protocol

Four studies utilized concentrated BMMNC harvested from the iliac crests of each patient as their SC treatment [2,9,14,35]. Zhao et al. used cultured autologous BMMNC harvested from the proximal femur of each patient as their SC treatment [8]. After bone marrow was harvested, in vitro expansion of the mesenchymal SC was performed for 2 weeks prior to reimplantation. SC treatment was performed in conjunction with CD for all clinical studies (Table 2).

#### Stem cell dose

SC dose was measured using a variety of methods - total mononucleated cell count (TMNCC), CD34+ cell count, number of fibroblast colony forming units (F-CFU) and total mesenchymal SC count (TMSCC). TMNCC is a measure of all the nucleated cells including mature nucleated cells and mesenchymal SC. CD34+ cells are a marker of hematopoeitic precursor cells and F-CFU are a measure of mesenchymal SC (each F-CFU is thought to arise from clonal expansion of mesenchymal SC). TMSCC is an approximate count of the number of SC and was used by Zhao et al. prior to reimplantation of cultured mesenchymal SC [8].

TMNCC was measured in 4 studies and ranged from 5 × 10^8^ to 19 × 10^8^ cells [2,9,14]. CD34+ cell counts were measured in two studies (1.9 × 10^7^ to 5 × 10^7^ cells) [2,14]. F-CFU counts were measured in two studies (mean F-CFU count of 1.76 × 10^3^ to 2.4 × 10^3^) [2,35]. TMSCC was reported in one study (2.0 × 10^6^ cells) [8] (Table 3).

**Table 3:** Stem cell dose

**Study**	**TMNCC**	**CD34+ cell count**	**F-CFU**	**TMSCC**
Hernigou et al. [35]	--	--	2.4 × 104	--
Wang et al. [9]	15.5 × 108 cells	--	--	--
Gangji et al. [2]	19 × 108 cells	1.9 × 107 cells	1.76 × 104	--
Sen et al. [14]	5 × 108 cells	5 × 107 cells	--	--
Zhao et al. [8]	--	--	--	2 × 106 cells

TMNCC = total mononucleated cell count, F-CFU = fibroblast colony forming units, TMSCC = total mesenchymal stem cell count.

#### Examined outcomes

Across studies a variety of patient reported outcomes were reported, including Harris Hip Score (HHS) (4 studies) [8,9,14,35], Lequesne index (1 study) [2], Western Ontario and McMaster Osteoarthritis index (WOMAC) (1 study) [2], and pain visual analog score (VAS) (1 study) [2]. Three studies examined progression to more advanced ARCO stage [2,8,9], and 3 studies examined volume of necrotic lesion by MRI [2,8,35]. Hip survivorship was measured in all 5 studies and was defined as conversion to THA (4 studies) [2,9,14,35], and conversion to THA or need for vascularized bone grafting (1 study) [8]. Two studies reported KM survival curves as part of their hip survivorship analysis [2,14].

### Clinical study results

#### Patient reported outcomes (5 studies)

Four of the studies reported HHS [8,9,14,35]. Zhao et al. found a statistically significant increase in HHS 60 months following SC therapy compared to CD only in patients with ARCO stage 1C/2B/2C (p < 0.05, raw score not reported), and a trend for ARCO stage 2A (p = 0.06, raw score not reported) [8]. Sen et al. found a statistically significant difference in HHS for the SC treated patients compared to CD only at 12 months follow up (83.65 ± 8.04 vs. 76.68 ± 13.86, p < 0.05) [14]. At 24 months, overall HHS was not significantly improved between the two groups (82.42 ± 9.63 vs. 77.39 ± 16.98, p = 0.09), but the pain and deformity domains of the HHS were still in favor of the SC therapy group (p < 0.05, raw score not reported) [14]. Wang et al. reported statistically significant (p < 0.05) improvements in HHS score at an average of 27.6 months post SC treatment for patients in ARCO stage 1/2A/2B/2C, from 90 ± 0.06 to 96 ± 0.06, 78.6 ± 1.02 to 92.5 ± 1.22, 68.2 ± 6.16 to 82.6 ± 8.23, and 67.8 ± 11.2 to 77.9 ± 15.15 respectively [9]. Hernigou et al. reported HHS on patients that had not progressed to having THA (420 of 534 hips) [35]. They reported HHS of 70 preoperatively and 88 post SC treatment at an average of 13 years follow up (no p value reported) [35].

Gangji et al. examined 3 patient reported outcomes: Lequesne index, VAS and WOMAC [2]. The SC treatment group had improved Lequesne index compared to CD group (SC group = 4.8 ± 1.8, CD group Lequesne index not reported, p = 0.03), and improved VAS (SC group = 20.8 ± 7.7, CD group VAS not reported, p = 0.009). WOMAC scores were not significantly different between the groups (raw scores not reported, p = 0.091) [2] (Table 4).

**Table 4:** Clinical study results for stem cell therapy

**Study**	**Patient reported outcomes**		**Progression**	**Lesion volume**	**Hip survivorship**
	Outcome				
Hernigou et al. [35]	HHS (13 years)	Improved compared to baseline score (88 vs 70)^		Decreased lesion volume (12 cm^3^ vs 26 cm^3^)^	17.6% conversion rate to THA at 13 years follow up
Wang et al. [9]	HHS	Improved compared to baseline score (83.7 ± 10.34 vs 71.2 ± 6.56)*	23.7% rate of progression to higher ARCO class		11.8% conversion to THA at 27.6 months
Gangji et al. [2]	Lequesne index	Improved compared to CD (SC group 4.8 ± 1.8, CD group NR)*	Decreased progression (23.1% vs 72.7%)*	Decreased lesion volume at 24 months (SC group volume decreased 42%, CD group volume decreased 1%)*, no improvement at 60 months (SC group volume decreased 42%, CD group volume decreased 22%)#	No improvement in hip survivorship (57.2 months vs 50.2 months)
	VAS pain	Improved compared to CD (SC group 20.8 ± 7.7, CD group NR)*			
	WOMAC	No improvement compared to CD (raw score NR)			
Sen et al. [14]	HHS (12 months)	Improved compared to CD (83.65 ± 8.04 vs 76.68 ± 13.86)*			Improved hip survivorship (51.85 ± 0.15 weeks vs. 46.62 ± 2.34 weeks)*
Zhao et al. [8]	HHS ARCO 1C,2B,2C (60 months)	Improved compared to CD (raw score NR)*	Decreased progression (3.8% vs. 22.7%)*	Decreased lesion volume in ARCO 2B (6.5% vs 13.3% of FH), and ARCO 2C (13.8% vs 29.3%)*	Decreased rate of conversion to THA/vascularized bone graft in the SC group compared to CD (3.7% vs. 22.7%)*

HHS = Harris Hip Score, ARCO = Association Research Circulation Osseous classification, VAS = visual analog scale pain score, WOMAC = Western Ontario and McMaster Universities Osteoarthritis index, ^ = no statistics reported, * = p < 0.05, # = p = 0.06, CD = core decompression, NR = not reported, FH = femoral head, THA = total hip arthroplasty.

#### Progression of avascular necrosis (3 studies)

Two of 3 studies found a significant decrease in the proportion of patients progressing to ARCO stage 3 or 4 following SC treatment compared to CD only treatment, 60 months post procedure (p < 0.05) [2,8]. Zhao et al. reported a decrease from 22.7% (CD group) to 3.7% (SC group) [8] and Gangji et al. reported a decrease from 72.7% (CD group) to 23.1% (SC group) [2]. Gangji et al. also performed a KM survival analysis with progression to ARCO stage 3 as the end point, and found a significantly longer (p < 0.05) time to progression to ARCO stage 3 in the SC group compared to CD [52.2 months (43.35 - 60.96, 95% confidence interval (CI)) vs 26.5 months (13.2 - 39.74, 95% CI)] [2]. In their case series, Wang et al. reported a 22% rate of progression to higher ARCO stage (for hips in ARCO stage 1 or 2 before SC treatment) at an average follow up of 27 months [9] (Table 4).

#### Volume of necrotic lesion (3 studies)

Zhao et al. reported a significant decrease in lesion volume in SC treated hips compared to CD only treated hips with a pretreatment ARCO stage of 2B and 2C (ARCO 2B = 6.5% vs 13.3% of FH, ARCO 2C = 13.8% vs 29.3% of FH, p < 0.05) at 60 months post treatment [8]. Gangji et al. reported a significant decrease in lesion volume after SC treatment compared to CD only at 24 months follow up (SC group volume decreased 42%, CD group volume decreased 1%, p < 0.05), and a trend towards decreased lesion volume at 60 months (SC group volume decreased 42%, CD group volume decreased 22%, p = 0.06) [2]. Hernigou et al. reported on lesion volume in 371 of 534 hips in their case series [35]. They found a decrease in lesion size from 26 cm^3^ to 12 cm^3^ at an average of 12 years follow up (no statistical analysis reported) [35] (Table 4).

#### Hip survivorship (5 studies)

Four studies examined conversion rate to THA [2,9,14,35]. Sen et al. performed KM hip survival analysis and they found the SC treatment group to have significantly longer hip survival compared to CD alone [51.85 weeks (51.54 - 52.15, 95% CI) vs 46.72 weeks (42.13 - 51.31, 95% CI), log rank test 4.44 p = 0.0351] [14]. Gangji et al’s nRCT found no significant difference in rate of conversion to THA between SC treatment and CD alone at 60 months of follow up (SC group: 14.3%, CD only group: 27.3%, p > 0.05) [2]. They also performed a KM survival analysis and found no significant difference in time to THA between the two groups [mean time to THA - SC group: 57.2 months (53.48–60.97 95% CI), CD only group: 50.2 months (40.24–60.13 95% CI), log rank test p = 0.42] [2]. Hernigou et al. found a 17.6% conversion rate in their series over an average follow up of 13 years [35], and Wang et al. found a conversion rate of 12% over an average follow up of 27 months [9].

Zhao et al. found a significant decrease in rate of conversion to THA or need for vascularized bone grafting in the SC group compared to CD alone (3.7% vs. 22.7%, p < 0.05) [8] (Table 4).

## Discussion

This review systematically examined the current literature on SC therapy for the treatment of early stage (precollapse) AVN of the FH including clinical and preclinical studies. Preclinical studies yielded encouraging results for treatment of AVN of the FH with SC. Although the source of SC varied among studies, SC treatment of AVN uniformly demonstrated improvements in osteogenesis and vascularization. All 11 studies showed positive effects with respect to bone formation in groups treated with SC. Furthermore, reported X-ray, SPECT, CT and MR outcomes from all studies favoured the SC treatment group. Bone marrow was the most common source of SC but other sources such as adipose and dental pulp were identified. SC isolated from dental pulp represents a relatively new treatment option with noteworthy potential for use in orthopaedics [4]. Adipose derived SC are another potential alternative to SC from bone marrow. Advantages of adipose derived SC include abundance, ease of isolation, rapid expansion, and multipotency [10].

Despite positive results, relevance of animal models described in preclinical studies should be considered. Corticosteroid and liquid nitrogen induced AVN of the FH are widely recognized means for induction of AVN in numerous animal models; both lead to ischemic conditions within the FM and eventual osseous infarction producing changes phenotypically similar and clinically relevant to human disease [36]. Some studies, however, have addressed the significance of larger animal models, particularly with respect to translational medicine, as they may better replicate conditions in human AVN. An ovine model of AVN of the FH may better reflect articulation in early-stage human AVN as compared to other disease models [4]. For liquid nitrogen induced disease, the bone defect produced, which is non-negligible in animals with small diameter FHs, has been proposed as a limitation due to its absence from human pathology. This has led some researchers to reject use of this method on rabbits [37].

Results of clinical studies were also encouraging. In our review, the clinical studies used CD as a means for implanting SC directly into the necrotic region of the FH, in the form of a cell suspension. CD works by decreasing intra-osseous pressure and improving circulation and vascularization [9]. Used alone, however, CD exhibits inconsistent outcomes including poor lesion reconstruction, ultimately leading to FH collapse [9,14]. The progression of AVN of the FH occurs in consequence of a limited capacity for articular tissue self-repair [3,11,14], including decreased osteogenesis [11,14] and vascularization [3]. This may occur as a result of inadequate numbers of progenitor cells in the proximal femur of patients with AVN of the FH [38]. It is thought that SC implanted into the necrotic region of the FH work to repopulate the low numbers of progenitor cells [20]. Pluripotent, mesenchymal SC differentiate into various cell types, namely osteoblasts, thereby improving repair mechanisms and potentially reversing damage to bone [11,12,14]. In addition to directly increasing bone formation by differentiating into osteoblasts, it is hypothesized that mesenchymal SC have an indirect effect by the expression of cytokines which influence osteogenesis and neovascularization [39,40]. In general, clinical studies reported improvements in patient reported outcomes for those treated with SC; notably, the HHS. Similarly, studies that examined progression to more advanced disease, and lesion volume reported improvements for the SC treatment group. Participants treated with SC did not experience consistent improvements in hip survivorship across studies. None of the studies using a comparative group found worse outcomes for SC treatment.

Considerable variations and inconsistent reporting among clinical studies were observed regarding the dose of SC, etiology of AVN, lesion size, and severity/classification of disease making comparisons between studies challenging. However, there are currently limited numbers of clinical studies addressing SC therapy for treatment of AVN of the FH, and even fewer addressing early-stage disease and administration of SC by CD. Accordingly, we were unable to perform meta-analysis on study results. Quantitative assessment will be a prerequisite to making definitive conclusions on vital therapy-related factors such as SC dose and quality.

Standardization of SC dose has proven difficult due to a lack of uniformly accepted, reliable cell markers which can be used to identify mesenchymal SC [39]. However, the dose of SC used has been reported to impact disease outcome [7]. Both SC dose and quality are also known to affect their clinical potential. Density of SC transplanted to the necrotic FH was shown to affect the rate of osteogenesis, and thereby bone repair [12]. Quality of transplanted cells affects their proliferative capacity [41]. Prior to routine use of combined SC/CD therapy, defined standards of SC dose and quality, such as CD34+ or CFU counts [42], will likely have to be set in order to accurately evaluate the effect of each therapy. However, as a result of presently observed inconsistencies, and a paucity of studies in this area, further research, examining both SC dose and quality will be prerequisite to routine clinical use of this therapy.

Though not specifically addressed by studies assessed in this review, the impact on treatment outcome of whether cells were derived from a concentrate or a culture may also represent an area for future research. Pre-clinical and clinical studies included examples of both concentrated and cultured cells. Concentrated cells contain all cells and cell types present in the tissue from which they have been derived, not only SC. Concentrated BMMNC from bone marrow aspirate contain hematopoietic progenitor cells, lymphocytes, leucocytes, in addition to non-hematopoietic cells including MSC, EPC, embryonic-like SC expressing pluripotent markers, and other multipotent or committed cells [43]. Cultured cells, conversely, represent an isolated pool of SC. The comparative regenerative capacity of concentrated vs. cultured cells remains unclear. Despite positive results observed for both treatments within this review, other studies explicitly assessing differences between the two have displayed mixed findings. Use of pure, cultured MSC led to greater improvements in ischemic limb diseases, compared to concentrated BMMNC, in both human and rat models [44,45]. Alternatively, BMMNC use displayed beneficial outcomes in treatment of spinal cord injury when compared to MSC [46]. Cost and feasibility must also be considered when selecting an appropriate treatment. Indeed, cultured cells require greater preparation times and are associated with increased cost [44,47]. Ultimately, the outcomes of concentrated vs. cultured cells should be assessed for the specific treatment of AVN of the FH in order to develop future robust clinical guidelines for cellular intervention in this disease.

Etiologic risk factors of AVN are also known to significantly affect treatment outcomes [2]. It has been demonstrated that the capacity for SC to differentiate into the necessary osteogenic cells for bone repair and remodeling is limited in patients with alcohol and steroid induced AVN of the FH due to differences in the ischemic environment [12,48]. The size of the osteonecrotic lesion is also known to affect overall patient outcome no matter the method of treatment used [8,49]. Future studies should aim to use the same AVN classification system as well as account for AVN etiology and lesion size as potential confounding variables.

Several other reviews [18,38,50-53] have been published discussing the use of SC for the treatment of AVN of the femoral head. However to our knowledge, ours is the first systematic review that includes data from several recently published clinical trials [2,8,14]. Our review included data from over 700 hips, more than previously published reviews. Additionally, our review included both clinical and pre-clinical studies, furthering the breadth of our review. A limitation of any systematic review is in the quality of the papers available for review. Clinical studies included in our review did not provide sample size and power calculations. Preclinical studies did not always use a classification system to identify stage of AVN of the FH. There were a limited number of comparative trials, and only two RCTs. We included all types of clinical studies, potentially introducing confounding and selection bias. We felt that inclusion of these studies would provide a more comprehensive review of the literature surrounding this topic. Furthermore, meta-analysis was not performed due to the limited number of comparative trials and variable methodology employed in the studies.

## Conclusions

AVN of the FH primarily affects younger, working age individuals and thus leads to increased morbidity and functional disability in this population [13,30]. Treatments aimed at halting or delaying progression of disease would provide a welcome alternative to those faced with progression to joint collapse and hip replacement surgery. Combining CD, the most widely used treatment for AVN of the FH to date [30], with SC, could result in a novel long lasting hip preserving treatment option. Pre-clinical studies have demonstrated the potential of SC for reversing the debilitating damage to the femoral head associated with AVN *in vitro*. Current clinical studies have suggested beneficial effects on patient reported outcomes, but definitive conclusions regarding hip survival and disease progression cannot be made. Further, more refined clinical studies are needed to establish the effectiveness of SC treatment in AVN of the FH. Quantitative studies including meta-analyses aimed at addressing SC dose and quality standards will be necessary to make fundamental treatment-related deductions. The effects of concentration and culture based preparatory methods for SC therapy should be compared in the context of treating AVN of the FH in order to establish suitable protocols. Demonstrated improvement in hip survivorship is prerequisite to the future of this treatment.

## Abbreviations

AVN: Avascular necrosis; FH: Femoral head; SC: Stem cells; THA: Total hip arthroplasty; CD: Core decompression; HHS: Harris Hip Score; CT: Computed tomography; MRI: Magnetic resonance imaging; SPECT: Single photon emission computed tomography; RCT: Randomized controlled trial; nRCT: Nonrandomized controlled trial; ARCO: Association Research Circulation Osseous classification; TMNCC: Total mononucleated cell count; TMSCC: Total mesenchymal stem cell count; WOMAC: Western Ontario and McMaster Universities Osteoarthritis index; VAS: Visual analogue scale pain score; BMMNC-MSC: mesenchymal stem cells obtained post BMMNC culture; hIDPSC: Human immature dental pulp stem cells; ADSC: Adipose derived stem cells; PBSC: Peripheral blood stem cells; EPC: Endothelial progenitor cells; cBMMNC: Cryoperserved bone marrow derived mononuclear cells; F-CFU: Fibroblast colony forming units.

## Competing interests

There are no competing interests for any author of this study.

## Authors’ contributions

RL contributed to the design of the study, performed collection and assembly of data, analysis and interpretation of data and drafting and critical revision of the article. RG conceived and designed the study, drafted and critically revised the article, and gave final approval of the article. HE performed data collection and assembly and drafting of the article. SM performed data collection and assembly and drafting of the article. NM participated in study design and coordination, revised and gave final approval of the article. All authors read and approved the final manuscript.

## Role of the funding source

No external funding was provided for the purposes of this study.

## Pre-publication history

The pre-publication history for this paper can be accessed here:

http://www.biomedcentral.com/1471-2474/15/156/prepub

## References

[B1] Yoshioka T Mishima H Akaogi H Sakai S Li M Ochiai N Concentrated autologous bone marrow aspirate transplantation treatment for corticosteroid-induced osteonecrosis of the femoral head in systemic lupus erythematosus Int Orthop 2011 35 823 829 doi:10.1007/s00264-010-1048-y 10.1007/s00264-010-1048-y 20512330 PMC3103953

[B2] Gangji V De Maertelaer V Hauzeur J-P Autologous bone marrow cell implantation in the treatment of non-traumatic osteonecrosis of the femoral head: Five year follow-up of a prospective controlled study Bone 2011 49 1005 1009 doi:10.1016/j.bone.2011.07.032 10.1016/j.bone.2011.07.032 21821156

[B3] Lee H-S Huang G-T Chiang H Chiou L-L Chen M-H Hsieh C-H Jiang C-C Multipotential mesenchymal stem cells from femoral bone marrow near the site of osteonecrosis Stem Cells 2003 21 190 199 doi:10.1634/stemcells.21-2-190 10.1634/stemcells.21-2-190 12634415

[B4] Feitosa M-L-T Fadel L Beltrão-Braga P-C-B Wenceslau C-V Kerkis I Kerkis A Birgel Júnior E-H Martins J-F Martins Ddos S Miglino M-A Ambrósio C-E Successful transplant of mesenchymal stem cells in induced osteonecrosis of the ovine femoral head: preliminary results Acta Cir Bras 2010 25 416 422 10.1590/S0102-86502010000500006 20877951

[B5] Matsuya H Kushida T Asada T Umeda M Wada T Iida H Regenerative effects of transplanting autologous mesenchymal stem cells on corticosteroid-induced osteonecrosis in rabbits Mod Rheumatol 2008 18 132 139 doi:10.1007/s10165-008-0023-6 10.3109/s10165-008-0023-6 18288561

[B6] Sun Y Feng Y Zhang C The effect of bone marrow mononuclear cells on vascularization and bone regeneration in steroid-induced osteonecrosis of the femoral head Joint Bone Spine 2009 76 685 690 doi:10.1016/j.jbspin.2009.04.002 10.1016/j.jbspin.2009.04.002 19576836

[B7] Hernigou P Beaujean F Treatment of osteonecrosis with autologous bone marrow grafting Clin Orthop Relat Res 2002 405 14 23 12461352 10.1097/00003086-200212000-00003

[B8] Zhao D Cui D Wang B Tian F Guo L Yang L Liu B Yu X Treatment of early stage osteonecrosis of the femoral head with autologous implantation of bone marrow-derived and cultured mesenchymal stem cells Bone 2012 50 325 330 doi:10.1016/j.bone.2011.11.002 10.1016/j.bone.2011.11.002 22094904

[B9] Wang B-L Sun W Shi Z-C Zhang N-F Yue D-B Guo W-S Xu S-Q Lou J-N Li Z-R Treatment of nontraumatic osteonecrosis of the femoral head with the implantation of core decompression and concentrated autologous bone marrow containing mononuclear cells Arch Orthop Trauma Surg 2010 130 859 865 doi:10.1007/s00402-009-0939-0 10.1007/s00402-009-0939-0 19621230

[B10] Abudusaimi A Aihemaitijiang Y Wang Y-H Cui L Maimaitiming S Abulikemu M Adipose-derived stem cells enhance bone regeneration in vascular necrosis of the femoral head in the rabbit J Int Med Res 2011 39 1852 1860 10.1177/147323001103900528 22117986

[B11] Hang D Wang Q Guo C Chen Z Yan Z Treatment of Osteonecrosis of the Femoral Head with VEGF (165) Transgenic Bone Marrow Mesenchymal Stem Cells in Mongrel Dogs Cells Tissues Organs 2012 195 6 495 506 doi:10.1159/000329502 10.1159/000329502 22056983

[B12] Yan Z Hang D Guo C Chen Z Fate of mesenchymal stem cells transplanted to osteonecrosis of femoral head J Orthop Res 2009 27 442 446 doi:10.1002/jor.20759 10.1002/jor.20759 18925660

[B13] Li Z-H Liao W Cui X-L Zhao Q Liu M Chen Y-H Liu T-S Liu N-L Wang F Yi Y Shao N-S Intravenous transplantation of allogeneic bone marrow mesenchymal stem cells and its directional migration to the necrotic femoral head Int J Med Sci 2011 8 74 83 21234272 10.7150/ijms.8.74 PMC3020395

[B14] Sen R-K Tripathy S-K Aggarwal S Marwaha N Sharma R-R Khandelwal N Early results of core decompression and autologous bone marrow mononuclear cells instillation in femoral head osteonecrosis: a randomized control study J Arthroplasty 2012 27 679 686 doi:10.1016/j.arth.2011.08.008 10.1016/j.arth.2011.08.008 22000577

[B15] Yan Z-Q Chen Y-S Li W-J Yang Y Huo J-Z Chen Z-R Shi J-H Ge J-B Treatment of osteonecrosis of the femoral head by percutaneous decompression and autologous bone marrow mononuclear cell infusion Chin J Traumatol 2006 9 3 7 16393508

[B16] Gangji V Hauzeur J-P Matos C De Maertelaer V Toungouz M Lambermont M Treatment of osteonecrosis of the femoral head with implantation of autologous bone-marrow cells. A pilot study J Bone Joint Surg Am 2004 86-A 1153 1160 15173287 10.2106/00004623-200406000-00006

[B17] Song H-J Lan B-S Cheng B Zhang K-F Yan H-W Wang W-Z Gao Z-Q Treatment of early avascular necrosis of femoral head by small intestinal submucosal matrix with peripheral blood stem cells Transplant Proc 2011 43 2027 2032 doi:10.1016/j.transproceed.2010.12.060 10.1016/j.transproceed.2010.12.060 21693320

[B18] Gangji V Hauzeur J-P Cellular-based therapy for osteonecrosis Orthop Clin North Am 2009 40 213 221 doi:10.1016/j.ocl.2008.10.009 10.1016/j.ocl.2008.10.009 19358906

[B19] Gangji V Toungouz M Hauzeur J-P Stem cell therapy for osteonecrosis of the femoral head Expert Opin Biol Ther 2005 5 437 442 doi:10.1517/14712598.5.4.437 10.1517/14712598.5.4.437 15934823

[B20] Hernigou P Poignard A Manicom O Mathieu G Rouard H The use of percutaneous autologous bone marrow transplantation in nonunion and avascular necrosis of bone J Bone Joint Surg (Br) 2005 87 896 902 doi:10.1302/0301-620X.87B7.16289 15972899 10.1302/0301-620X.87B7.16289

[B21] Gangji V Hauzeur J-P Treatment of osteonecrosis of the femoral head with implantation of autologous bone-marrow cells. Surgical technique J Bone Joint Surg Am 2005 87 Suppl 1 106 112 doi:10.2106/JBJS.D.02662 15743852 10.2106/JBJS.D.02662

[B22] Hernigou P Daltro G Filippini P Mukasa M-M Manicom O Percutaneous implantation of autologous bone marrow osteoprogenitor cells as treatment of bone avascular necrosis related to sickle cell disease Open Orthop J 2008 2 62 65 doi:10.2174/1874325000802010062 10.2174/1874325000802010062 19478932 PMC2687112

[B23] Kawate K Yajima H Ohgushi H Kotobuki N Sugimoto K Ohmura T Kobata Y Shigematsu K Kawamura K Tamai K Takakura Y Tissue-engineered approach for the treatment of steroid-induced osteonecrosis of the femoral head: transplantation of autologous mesenchymal stem cells cultured with beta-tricalcium phosphate ceramics and free vascularized fibula Artif Organs 2006 30 960 962 doi:10.1111/j.1525-1594.2006.00333.x 10.1111/j.1525-1594.2006.00333.x 17181837

[B24] Pak J Autologous adipose tissue-derived stem cells induce persistent bone-like tissue in osteonecrotic femoral heads Pain Physician 2012 15 75 85 22270740

[B25] Peng J Wen C Wang A Wang Y Xu W Zhao B Zhang L Lu S Qin L Guo Q Dong L Tian J Micro-CT-based bone ceramic scaffolding and its performance after seeding with mesenchymal stem cells for repair of load-bearing bone defect in canine femoral head J Biomed Mater Res Part B Appl Biomater 2011 96 316 325 doi:10.1002/jbm.b.31770 21210512 10.1002/jbm.b.31770

[B26] Xiao Z-M Jiang H Zhan X-L Wu Z-G Zhang X-L Treatment of osteonecrosis of femoral head with BMSCs-seeded bio-derived bone materials combined with rhBMP-2 in rabbits Chin J Traumatol 2008 11 165 170 18507947 10.1016/s1008-1275(08)60035-8

[B27] Xu M Peng D Mesenchymal stem cells cultured on tantalum used in early-stage avascular necrosis of the femoral head Med Hypotheses 2011 76 199 200 doi:10.1016/j.mehy.2010.09.028 10.1016/j.mehy.2010.09.028 20970260

[B28] Yamasaki T Yasunaga Y Ishikawa M Hamaki T Ochi M Bone-marrow-derived mononuclear cells with a porous hydroxyapatite scaffold for the treatment of osteonecrosis of the femoral head: a preliminary study J Bone Joint Surg (Br) 2010 92 337 341 doi:10.1302/0301-620X.92B3.22483 20190302 10.1302/0301-620X.92B3.22483

[B29] Song H-J Peripheral Blood Stem Cell Transplantation for Ischemic Femoral Head Necrosis Transplant Proc 2010 42 7 2824 doi: 10.1016/j.transproceed.2010.02.077 10.1016/j.transproceed.2010.02.077 20620538

[B30] Wen Q Ma L Chen Y-P Yang L Luo W Wang X-N Treatment of avascular necrosis of the femoral head by hepatocyte growth factor-transgenic bone marrow stromal stem cells Gene Ther 2008 15 1523 1535 doi:10.1038/gt.2008.110 10.1038/gt.2008.110 18633448

[B31] Sun Y Feng Y Zhang C Cheng X Chen S Ai Z Zeng B Beneficial effect of autologous transplantation of endothelial progenitor cells on steroid-induced femoral head osteonecrosis in rabbits Cell Transplant 2011 20 233 243 doi:10.3727/096368910X522234 10.3727/096368910X522234 20719092

[B32] Xie X-H Wang X-L He Y-X Liu Z Sheng H Zhang G Qin L Promotion of bone repair by implantation of cryopreserved bone marrow-derived mononuclear cells in a rabbit model of steroid-associated osteonecrosis Arthritis Rheum 2012 64 1562 1571 doi:10.1002/art.34525 10.1002/art.34525 22544527

[B33] Wen Q Jin D Zhou C-Y Zhou M-Q Luo W Ma L HGF-transgenic MSCs can improve the effects of tissue self-repair in a rabbit model of traumatic osteonecrosis of the femoral head PLoS One 2012 7 e37503 doi:10.1371/journal.pone.0037503 10.1371/journal.pone.0037503 22629409 PMC3357393

[B34] Aimaiti A Saiwulaiti Y Saiyiti M Wang Y-H Cui L Yusufu A Therapeutic effect of osteogenically induced adipose derived stem cells on vascular deprivation-induced osteonecrosis of the femoral head in rabbits Chin J Traumatol 2011 14 215 220 21801665

[B35] Hernigou P Poignard A Zilber S Rouard H Cell therapy of hip osteonecrosis with autologous bone marrow grafting Indian J Orthop 2009 43 40 45 doi:10.4103/0019-5413.45322 10.4103/0019-5413.45322 19753178 PMC2739495

[B36] Hernigou P Beaujean F Lambotte J-C Decrease in the mesenchymal stem-cell pool in the proximal femur in corticosteroid-induced osteonecrosis J Bone Joint Surg (Br) 1999 81 349 355 10.1302/0301-620X.81B2.8818 10204950

[B37] Jones E Yang X Mesenchymal stem cells and bone regeneration: current status Injury 2011 42 562 568 doi:10.1016/j.injury.2011.03.030 10.1016/j.injury.2011.03.030 21489533

[B38] Rackwitz L Eden L Reppenhagen S Reichert JC Jakob F Walles H Pullig O Tuan R-S Rudert M Nöth U Stem cell- and growth factor-based regenerative therapies for avascular necrosis of the femoral head Stem Cell Res Ther 2012 3 7 doi:10.1186/scrt98 10.1186/scrt98 22356811 PMC3340551

[B39] Suh K-T Kim S-W Roh H-L Youn M-S Jung J-S Decreased osteogenic differentiation of mesenchymal stem cells in alcohol-induced osteonecrosis Clin Orthop Relat Res 2005 431 220 225 15685079 10.1097/01.blo.0000150568.16133.3c

[B40] Mont M-A Jones L-C Hungerford D-S Nontraumatic osteonecrosis of the femoral head: ten years later J Bone Joint Surg Am 2006 88 1117 1132 doi:10.2106/JBJS.E.01041 10.2106/JBJS.E.01041 16651589

[B41] Jones L-C Hungerford D-S Osteonecrosis: etiology, diagnosis, and treatment Curr Opin Rheumatol 2004 16 4343 4449 10.1097/01.moo.0000127829.34643.fd 15201609

[B42] Hauzeur J-P Gangji V Phases 1–3 clinical trials using adult stem cells in osteonecrosis and nonunion fractures Stem Cells Int 2010 2010 410170 doi:10.4061/2010/410170 21048847 10.4061/2010/410170 PMC2964482

[B43] Cuende N Rico L Herrera C Concise Review: Bone Marrow Mononuclear Cells for the Treatment of Ischemic Syndromes: Medicinal Product or Cell Transplantation? Stem Cells Trans Med 2012 1 5 403 408 10.5966/sctm.2011-0064 PMC3659705 23197819

[B44] Iwase T Nagaya N Fujii T Itoh T Murakami S Matsumoto T Kangawa K Kitamura S Comparison of Angiogenic Potency Between Mesenchymal Stem Cells and Mononuclear Cells in a Rat Model of Hindlimb Ischemia Cardiovasc Res 2005 66 543 551 10.1016/j.cardiores.2005.02.006 15914119

[B45] Lu D Chen B Liang Z Deng W Jiang Y Li S Xu J Wu Q Zhang Z Xie B Chen S Comparison of Bone Marrow Mesenchymal Stem Cells with Bone Marrow-Derived Mononuclear Cells for Treatment of Diabetic Critical Limb Ischemia and Foot Ulcer: A Double-Blind, Randomized Controlled Trial Diabetes Res Clin Pract 2011 92 1 26 36 10.1016/j.diabres.2010.12.010 21216483

[B46] Samdani A-F Paul C Betz R-R Fischer I Neuhuber B Transplantation of Human Marrow Stromal Cells and Mono-Nuclear Bone Marrow Cells into the Injured Spinal Cord: A Comparative Study Spine (Phila Pa 1976) 2009 34 24 2605 2612 10.1097/BRS.0b013e3181bdca87 19881401

[B47] Ikebe C Suzuki K Mesenchymal Stem Cells for Regenerative Therapy: Optimization of Cell Preparation Protocols Biomed Res Int 2014 2014 951512 24511552 10.1155/2014/951512 PMC3912818

[B48] Jones K-B Seshadri T Krantz R Keating A Ferguson P-C Cell-based therapies for osteonecrosis of the femoral head Biol Blood Marrow Transplant 2008 14 1081 1087 doi:10.1016/j.bbmt.2008.06.017 10.1016/j.bbmt.2008.06.017 18804037

[B49] Sen R-K Management of avascular necrosis of femoral head at pre-collapse stage Indian J Orthop 2009 43 6 16 doi:10.4103/0019-5413.45318 10.4103/0019-5413.45318 19753173 PMC2739499

[B50] Schallmoser K Bartmann C Wagner W Replicative Senescence-Assosiated Gene Expression Changes in Mesenchymal Stromal Cells are Similar under Different Culture Conditions Haematologica 2010 95 6 867 874 10.3324/haematol.2009.011692 20053868 PMC2878782

[B51] Pamphilon D Mijovic A Storage of Hemopoietic Stem Cells Asian J Transfus Sci 2007 1 2 71 76 10.4103/0973-6247.33848 21938237 PMC3168124

[B52] Boss J-H Misselvich I Osteonecrosis of the Femoral Head of Laboratory Animals: The Lessons Learned from a Comparative Study of Osteonecrosis in Man and Experimental Animals Vet Pathol 2003 40 4 345 354 10.1354/vp.40-4-345 12824505

[B53] Wang C Wang J Zhang Y Yuan C Liu D Pei Y Li X Wu Z Li Y Guo Z A Canine Model of Femoral Head Osteonecrosis Induced by an Ethanol Injection Navigated by a Novel Template Int J Med Sci 2013 10 11 1451 1458 10.7150/ijms.6314 24046517 PMC3775100

